# Management of an Acute Airway Obstruction Due to Tracheal Carcinoma in a Patient With Severe Glottic Stenosis

**DOI:** 10.7759/cureus.33203

**Published:** 2023-01-01

**Authors:** Lentiona V Basiari, Maria C Michali, Ioannis D Komnos, Eleni V Litsou, Georgios V Psychogios

**Affiliations:** 1 Department of Otorhinolaryngology, Head, and Neck Surgery, University Hospital of Ioannina, Ioannina, GRC

**Keywords:** dyspnea, airway obstruction, second primary malignancy, endotracheal debulking, surgery, squamous cell carcinoma, tracheal malignancy

## Abstract

In this paper, we present the case of acute airway obstruction due to tracheal carcinoma in a patient with glottic stenosis due to previously treated laryngeal carcinoma. Because of severe dyspnea from the obstructive endotracheal mass, tracheotomy under local anesthesia was immediately performed. Intubation with pediatric size (I.D. 4.5 mm) cuffed endotracheal tube was performed by the surgeon through tracheostomy under endoscopic visualization. Blakesley forceps and electrocautery were used for tumor debulking. Postoperatively there were no complications and the patient was discharged after four days. The histopathology report showed a squamous cell carcinoma. The tumor board decided on adjuvant chemoradiotherapy for the treatment of the patient.

## Introduction

Primary malignant neoplasms of the trachea are very rare. With an incidence of 0.1 per 100,000 persons per year, these tumors account for 0.2% of respiratory tract malignancies and approximately 0.02-0.04% of all malignancies [1]. This rarity limits our knowledge of tracheal carcinomas and makes them a diagnostic and therapeutic challenge. Although extremely rare, these tumors present variable histological and clinical characteristics [2]. They can arise from the respiratory epithelium, mesenchymal structures, and the salivary glands of the tracheal mucosa. The two most common histological types that account for about two-thirds of adult primary tracheal carcinomas are squamous cell carcinoma (SCC) and adenoid cystic carcinoma (ACC). The remaining one-third is part of a heterogeneous group that includes mucoepidermoid carcinoma, adenocarcinoma, sarcoma, chondrosarcoma, neuroendocrine tumors, and other rare types [3,4]. SCC is most frequent among smokers in contrast to ACC, which is the most common tracheal malignancy in non-smokers [5]. SCC of the trachea can present as a second primary malignancy related to SCC of the lung, larynx, and oropharynx, which highlights the importance of active surveillance and follow-up in these patients. Tracheal tumors can be life-threatening by causing airway obstruction, fatal hemorrhage from locoregional invasion, and distant metastatic disease. In most cases, there is a delay in diagnosis, and patients present with advanced, unresectable diseases [6]. Non-specific complaints of cough, stridor, dyspnea on exertion, and wheezing can lead to the misdiagnosis of adult-onset asthma or even chronic obstructive pulmonary disease (COPD) [7]. This misdiagnosis often delays proper treatment. When possible, surgery with resection of the involved segment of the trachea followed by radiotherapy is the treatment of choice. In other cases of advanced disease and contraindications to surgery, palliative interventions can be done with techniques of endotracheal debulking or endotracheal stenting followed by chemoradiotherapy. In this paper, we aim to present the management of acute life-threatening airway obstruction due to tracheal carcinoma in a patient with glottic stenosis.

## Case presentation

**Figure 1:**
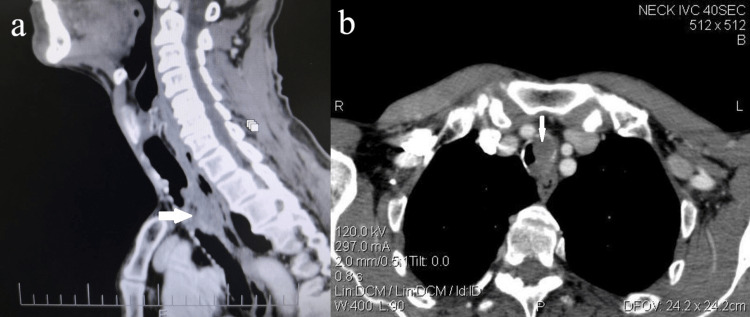
(a) Sagittal view of CT revealing the extension of the tumor (white arrow). (b) Axial view of CT revealing the extent of obstruction of a tracheal lumen (white arrow). CT: computed tomography

A 67-year-old male patient presented to our emergency department with severe dyspnea and stridor at rest. During the last three months, he mentioned that he had been suffering from progressive dyspnea. In the past, he was a heavy smoker (40 pack years) and his BMI was 20 kg/m^2^. Five years ago, he was diagnosed with a T2 glottic SCC of the larynx and treated with transoral laser cordectomy, a temporary tracheostomy, and adjuvant radiotherapy (total dose of 66 Gy) elsewhere. Examination with a flexible endoscope showed severe stenosis of the glottis (75% of the airway between the vocal folds) due to postoperative adhesions, especially in the anterior commissure, and an endotracheal tumor with occlusion of about 85% of the trachea lumen. There was no evidence of malignancy in the larynx. Also, there were no palpable lymph nodes in the neck. The patient had a recent computed tomography (CT) scan that showed a large obstructive endotracheal mass arising from the posterior tracheal wall with dimensions of 2.4 x 1.4 x 4.5 cm, approximately, with no evidence of lymph node metastases or locoregional invasion (Figure 1a, 1b).

**Figure 2:**
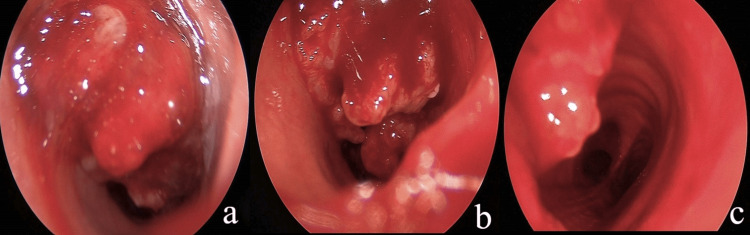
(a) Intraoperative endoscopic visualization of the tumor after tracheotomy was performed. (b) Intraoperative endoscopic visualization, from tracheostomy, of tumor extension inside the tracheal lumen. (c) Endoscopic visualization of the carina after partial tumor debulking.

The tumor distance from the glottis was approximately 4 cm, and the distance from the carina was approximately 4.5 cm. Because of severe dyspnea and low blood oxygen saturation, we immediately performed a tracheotomy under local anesthesia between the second and third tracheal rings (at the previous scar). Using a 45° rigid endoscope that was inserted from the tracheostomy, we managed to visualize all the tumors, which extended below the tracheostomy until a few cm above the carina (Figure 2a, 2b, 2c).

**Figure 3:**
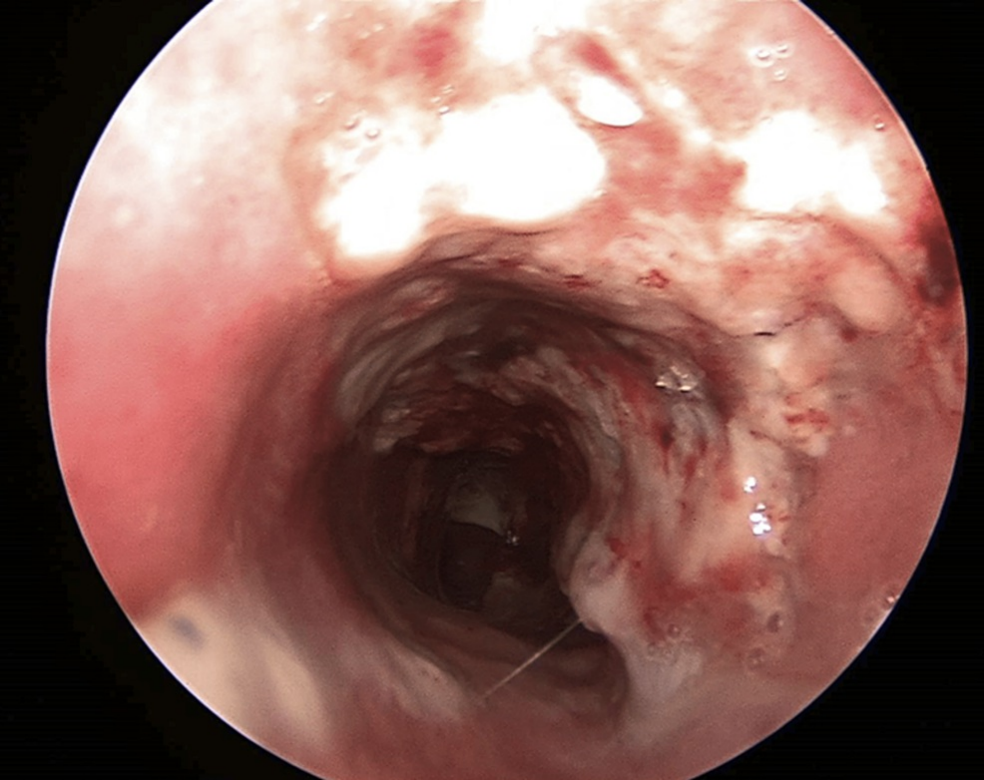
Postoperative endoscopic examination before patient discharge.

Then, under endoscopic visualization, intubation was performed with a pediatric-size (I.D. 4.5 mm) cuffed endotracheal tube from the surgeon through tracheostomy to avoid injury to the tumor and bleeding. General anesthesia was administered by the anesthesiologist. Debulking was performed with Blakesley forceps and bipolar electrocautery. Suction was used constantly to prevent blood from entering the lung and bronchi. Specimens were sent for histopathological examination. At the end of the operation, we placed an extended-length cuffed tracheostomy tube with an interior diameter of 8 mm. Postoperatively, there was no need for an intensive care unit. There wasn’t any complication or need for oxygen therapy. A postoperative endoscopic examination was performed (Figure 3), and the patient was discharged after four days.

The biopsy revealed an SCC with moderate-to-low differentiation. The tumor board decided on postoperative chemotherapy and radiation therapy for the patient after consultation. The patient decided to undergo chemoradiotherapy in another center and received a dose of 64 Gy of radiotherapy with concurrent chemotherapy. One year after surgery, the patient was alive with good local control of the disease but unfortunately presented with brain metastasis.

## Discussion

Primary tracheal carcinoma is the rarest malignancy of the airway, and this can be attributed to the role of local mucosa in immunosurveillance [8]. SCC is the most common histological type, affecting men more frequently in their sixth and seventh decades of life. It is strongly related to the use of tobacco and alcohol [7,9]. It usually manifests as an obstructive, exophytic endotracheal lesion. Clinical symptoms usually include dyspnea, wheezing, and stridor due to upper airway obstruction, as well as cough and hemoptysis due to mucosal irritation and ulceration. Hoarseness and signs of aspiration can also be present and might indicate recurrent laryngeal nerve invasion in some cases [7]. In our patient, the main symptom was progressive dyspnea at rest because the tracheal lumen had severe obstruction as noted on CT [10]. He also presented with hoarseness, which in our case, was related to his past history of transoral laser cordectomy and glottic stenosis, and not due to local invasion of the recurrent laryngeal nerves. It has been reported that approximately 40% of tracheal SCC are synchronous or metachronous second primary malignancies related to SCC of the lung, larynx, or oropharynx. ACC can also present as a primary malignancy but not as often as SCC [11]. Our patient had been treated for laryngeal SCC of the glottis five years earlier with transoral laser cordectomy, temporary tracheostomy, and adjuvant radiotherapy, and the tracheal lesion had a distance of 4 cm from the glottis. Tracheal cancer in our patient could be the case of a metachronous second primary malignancy of the trachea, assuming as an index tumor the SCC of the larynx. Patients with head and neck malignancies present an increased risk for the development of second primary malignancies due to the "field cancerization" concept which is related to genetic alterations and premalignant changes in the adjacent mucosa. SCC of the larynx is mostly associated with second primary malignancies of the lung and bronchi and rarely with second primary malignancies of the trachea as seen in our case [12]. Acute airway management in these patients can be extremely difficult and challenging.

For radiological evaluation, we used the CT of the neck and chest that the patient brought when he presented to the emergency department. CT is the most appropriate exam that provides information about the extension of the tumor, the depth of invasion, the invasion of adjacent structures, and the presence of lymphogenic or distant metastases. It can also provide excellent spatial resolution and has the ability to generate three-dimensional reconstructions, which allows for rapid evaluation of the extent of tracheal pathologies. Magnetic resonance imaging (MRI) is helpful in imaging tracheal compression or invasion by mediastinal masses or vascular anomalies and fluorodeoxyglucose positron emission tomography (FDG PET)/CT is reserved for staging patients with tracheobronchial malignancies. In our case, the carina was not involved, and there was no evidence of lymph node metastasis or invasion of adjacent organs. According to the CT, the extension was 4.5-5 cm in length, and more than 85% of the tracheal lumen was obstructed. In order to perform surgery with the goal of free margins of resection, it would be necessary to resect a large part of the trachea, including the previous tracheostomy scar, and in this case, an end-to-end anastomosis would present difficulty and excessive tension. In a previously irradiated trachea, this could lead to severe, life-threatening postoperative complications.

Debulking with interventional rigid bronchoscopy could be very risky and even fatal in our case. Endotracheal intubation in a patient with severe glottic stenosis and a high degree of endotracheal obstruction who already had severe dyspnea could be impossible and even dangerous, causing complete airway obstruction. Unsuccessful oral endotracheal intubation attempts might cause severe bleeding from the tumor, which wouldn’t be easy to control, leading to airway compromise and death. Hence, to manage the airway safely, we chose to perform a tracheotomy under local anesthesia and then proceed with intubation through it under direct endoscopic visualization. In this way, with the lung and bronchi protected, we performed debulking and managed to prevent airway obstruction and thus save the patient’s life.

## Conclusions

Tracheal tumors are rare malignancies and management of acute airway obstruction in these cases can be very challenging. CT is necessary for the diagnostic procedure and surgical resection is advocated as the procedure of choice for complete excision when it is possible. In this paper, we document a case of acute airway obstruction from a large endotracheal tumor which was not feasible to be totally resected. In cases like this, the aim is to save the patient’s life which can be achieved by performing tracheotomy and tumor debulking from the tracheostomy. After that, the patient could have the opportunity to undergo chemoradiotherapy and also have the chance of better overall survival.
